# The Metrology of a Rastered Spot of X Rays used in Security Screening

**DOI:** 10.6028/jres.119.021

**Published:** 2014-11-06

**Authors:** Lawrence T Hudson, Jack L Glover, Ronaldo Minniti

**Affiliations:** National Institute of Standards and Technology, Gaithersburg, MD 20899

**Keywords:** advanced imaging technology, air kerma, air kerma rate, backscatter, dosimetry, ionization chamber, rastered beam, security screening, swept beam, x rays

## Abstract

In recent times, ionizing radiation has been used around the world to screen persons for non-medical purposes, namely to detect bulk explosives or other contraband hidden on the body including materials not registered by metal detectors. In contrast to conventional transmission or projection imaging, backscatter and forward-scatter systems employ a “flying spot” of x rays and large-area detectors. A small spot is rastered across an individual and the Compton scatter signal collected by these detectors is quickly integrated and assigned to a pixel value in an image corresponding to the transient location of the small flying spot. These systems have been controversial due in part to possible radiation health risks, and lack of independent and accurate measurements of radiation exposures to the subjects, bystanders, and operators of such systems. In this paper we will outline the techniques and instrumentation used at the National Institute of Standards and Technology (NIST) to accurately determine the incident air kerma from a swept beam of x rays. We discuss in detail the response of a large-area free-air ionization chamber under the unusual temporal and spatial radiation fields delivered by commercial scanning systems and report typical values for air kerma levels as well as estimates of air kerma rates.

## 1. Introduction

In response to attempts by suicide bombers to ignite explosives during international airline flights, Advanced Imaging Technologies (AIT) were introduced at US airports. AIT devices detect both metallic and non-metallic threats concealed under clothing that cannot always be detected by electronic metal detectors. One class of AIT, x-ray backscatter imagers, has been in use since the 1990s in some countries for customs inspections; they also are used in diamond mines, prisons, and at military checkpoints. Their introduction at the aviation checkpoint was met with public controversy in part related to radiation dose to the traveling public, the accuracy of measuring dose given the unusual spatial and temporal characteristics of the source, and effectiveness to image and detect threats. The National Institute of Standards and Technology (NIST) has actively pursued the development of national and international consensus standards needed to accurately gauge both the imaging performance and radiation safety of AIT. Table 1 lists some of the most relevant standards and guides related to the metrology of x-ray AIT.

In the course of these standards development efforts, and in part to contribute to what was becoming a misinformed public dialog, NIST evaluated the recommended standard techniques for gauging *incident* air kerma (*i.e.*, without backscatter) from commercial x-ray AIT sources. Assuring the measurement accuracy of these rastered x-ray beams is important since this is the starting point in estimating individual organ doses and effective whole-body doses. In this paper we discuss how the x-ray backscatter systems produce images, and in that light we quantify the accuracy of the recommended dosimetric methods when applied to the metrology of a “flying spot” of x rays. We then estimate typical air-kerma rates from these systems. While the rates would be expected to follow the usual inverse square law with distance from the source, our measurements reveal that the air kerma levels themselves do not in general. We explain the observed distance dependencies which are relevant to modeling dose distributions throughout a body. The application of the documentary standards, recommendations, and guides in Table 1, and the understanding from the measurements and observations presented here, provide sufficient basis to conclude that the dosimetry of x-ray AIT produces results that are accurate at the few percent level and that agree well with simulations from first principles.

## 2. Concept of Operation

When studies of exposures were first undertaken [8] of x-ray backscatter systems that were designed for security screening of persons, the reported estimated dose levels were often met with incredulity. Whole-body (backscatter) images were being produced with conventional x-ray tubes operated at between 50 kV and 100 kV and with several milliamps of tube current, not too dissimilar from the parameter values used in medical x-ray devices. How then could such images impart doses that are thousands of times smaller than those experienced from typical medical x-ray procedures? Further, medical x-ray imaging of a person produces a deep dose, but because the backscatter image primarily employs x rays that had not penetrated very far into the subject, some assumed (wrongly) that the dose was shallow and “concentrated” in the skin. These and other early concerns and confusions are resolved by considering the x-ray physics and the techniques that are used to create and measure images produced by scattered x rays.

X-ray spectra from conventional x-ray tubes with applied potentials of 50 kV to 100 kV interact with human subjects primarily through Compton and Rayleigh scattering and photoelectric absorption, with an absorbed dose that decreases with penetration depth. Typically in security screening, at least the front and rear views are imaged, hence consideration of individual organ doses and whole-body effective doses is appropriate in this application and may be assessed using the same established protocols and guidelines as applied to medical applications.

**Fig. 1:**
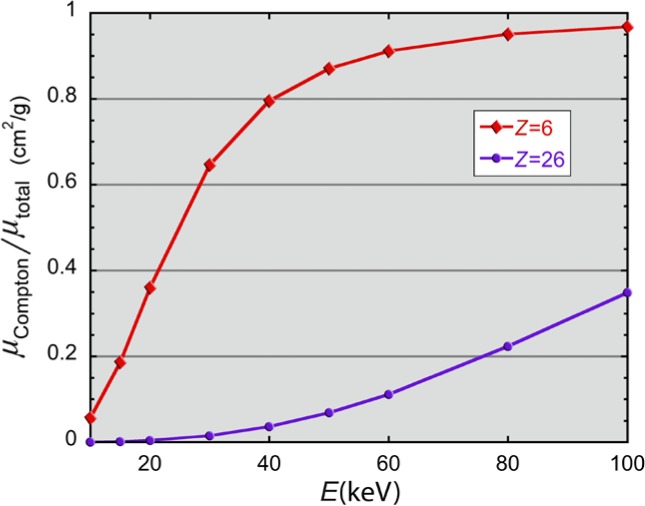
Ratio of Compton scattering mass attenuation coefficient to the sum of the mass attenuation coefficients of all processes, across the photon energy range used by commercial x-ray backscatter security scanners [9]. This is intended to illustrate the physical basis for the relative backscatter intensity from objects of low *Z* (*e.g*., carbon, *Z* = 6) and high *Z* (*e.g.*, iron, *Z* = 26), that in turn provides most of the contrast in a backscatter image.

X-ray backscatter image contrast follows from the dependence of the various x-ray interaction processes with the atomic number (*Z*) of the scattering material. Typically, only a few percent of the incident x rays contribute to a backscatter image. At a given photon energy and *Z*, the relative probability of scattering or absorption is the ratio of their cross sections. But to compare relative backscatter from objects of different *Z*, it is more helpful to plot the ratio of the Compton mass-attenuation coefficient to the total cross section. At these x-ray energies, the total cross section is the sum of inelastic Compton, elastic Rayleigh, and photoelectric absorption mass-attenuation coefficients. This ratio is shown in Fig. 1 as a function of x-ray energy for carbon (*Z* = 6) and iron (*Z* = 26) taken from entries in the NIST XCOM database [9]. The two choices of *Z* are intended as surrogates for low-*Z* materials, such as plastics, and high-*Z* materials, such as metals. In the case of the former, Compton scattering dominates and such objects appear relatively brighter in a backscatter image than high-*Z* materials where the photoelectric cross section dominates. (Note that edge effects can also assist in discerning overlapping materials of similar *Z*.) The abscissa axis covers the range of x-ray energies typically employed in the backscatter screening technique when applied to persons. At these energies, Rayleigh scattering is strongly forward directed and so would only contribute to a forward-scatter image; the use of such forward-directed images has been to detect items on the sides of an “x-ray shadow” of the body, supplementing backscatter detection of items on the front or back of a body.

**Fig. 2:**
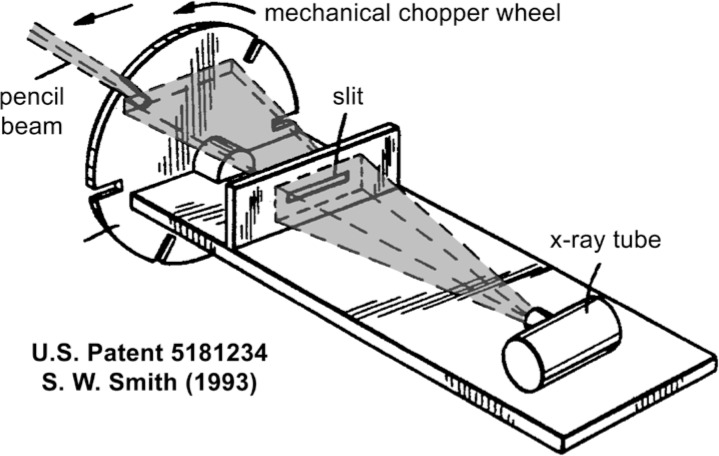
Annotated illustration from reference [10] showing the formation of a laterally-swept pencil beam of x rays. The vertical component of the scan is accomplished through translation, and sometimes also tilting, this entire assembly during the two-dimensional raster scan.

To understand how the backscattered x rays are used to produce an image, consider Fig. 2 taken from the first patent for such a system intended for security screening of persons [10]. A horizontal slit forms a fan beam that is itself further vignetted by a mechanical chopper wheel, producing a pencil beam that would appear to be swept laterally at a speed determined by the wheel rotational velocity. This entire assembly is then translated, and in some implementations also tilted, to achieve two-dimensional coverage of the subject to be scanned. X rays that are backscattered, and in some systems also forward scattered, from a subject are integrated by large-area detectors that either line the sides of the source cabinet or ride along with the source within the cabinet. The integral of the scattered signal is assigned to a pixel value in a two-dimensional image corresponding to the transient location of the flying spot. In the case of the backscattered image, the x rays that reach the detector have mostly passed through any clothing and have scattered from the near surface of the skin or from objects on the body of the person being scanned. Image resolution is determined primarily by the lateral dimensions of the small pencil beam; efficiency is obtained by collecting scattered x rays over a large solid angle. Since in practice persons being screened are required to divest themselves of pocket contents, jewelry, and overcoats, the task is to screen for *any* anomalies rather than the more difficult task of identification of specific threats; it also follows that image resolution is then not required to be as fine as in medical applications.

When x-ray backscatter systems were first introduced to the U.S. aviation checkpoint in 2007, security and proprietary concerns prevailed over transparency regarding the technical operating details of these systems. Independent attempts were made to estimate the dose to passengers from these systems. Unfortunately they were based upon assumptions that often led to incorrect conclusions and many conflicting claims. Over time, more details have become public and now researchers can model the radiation interactions from these security-screening systems with more definitive input values. Here we list some specifications from Annex 1 of a recent report evaluating the health effects of security scanners for passenger screening [7]. These values were provided by a manufacturer of airport security x-ray backscatter scanners and would of course differ by vendor. Our use here is illustrative, and will help facilitate comparisons to medical exposures.

Two general observations follow readily from Fig. 2 and Table 2. First, there is no “concentration” of the x-ray beam in either time or space; for many purposes, the metrology of a flying spot can be viewed as the metrology of a moving aperture in front of a conventional x-ray source, albeit in motion itself. Secondly, while two-dimensional medical images of subjects generally expose the entire image field for the duration of the exposure (often gauged by the product of beam current and time), during the 2.6 s scan of Table 1, the beam pass-by time on each point of the body is only 35 µs. At 5 mA, this would constitute an exposure of 175 nA·s. Medical exposures are generally well into the mA·s range, hence, coupled with the more modest image resolution requirement, one begins to appreciate how the reported absorbed dose estimates from security screening can reasonably be orders of magnitude below medical diagnostic screening. But of course, absolute metrology is required, so we next assess the effectiveness of the recommended dosimetric methods and instrumentation.

## 3. Instrumentation for the Metrology of a Rastered Spot of X Rays

Since the incident air kerma to be measured is generally only slightly higher than background levels, the standards and guidance documents in Table 1 generally recommend, for sensitivity, using a large-volume, free-air (thin-walled) ionization chamber (IC) with a high-quality electrometer with 0.1 pC resolution for the charge collection. Room-temperature semiconductor detectors can be used, though they exhibit a higher dependence upon incident x-ray energy, which is sometimes relevant to the transfer of calibration from laboratory to different sources in the field. Whatever the choice of instrument and read-out system, its response should be calibrated in a reference beam that closely approximates the x-ray spectrum of the security scanner being tested (or multiple references that bound the spectrum of interest). This in turn generally requires knowledge or measurement of the high-energy cutoff of the output spectrum and its half-value layer. The calibration should be traceable to an absolute realization of the radiometric quantity air kerma expressed in units of grays (Gy).

**Fig. 3:**
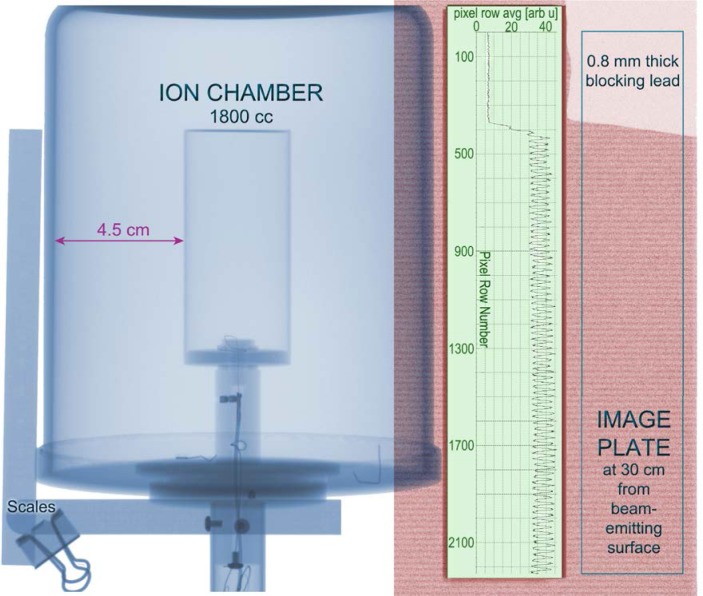
X-Ray image of a large-area ion chamber (left) proportionately scaled and superimposed upon an image acquired 30 cm from the beam-emitting surface of an AS&E SmartCheck X-ray Inspection System that employed an x-ray tube potential of 100 kV. External (clamped) scales are seen, establishing the distance between the cylindrical electrodes. The right image shows the relative signal of the rastered pencil beam of x rays as registered by an image plate detector; the upper right hand corner was blocked by lead to determine the background-signal level of the image plate. The vertical spatial variation in signal is plotted by averaging the pixel values in each row for the tall rectangular region of interest shown.

Several studies, including this report, have employed a cylindrical ionization chamber model Radcal 10X5-1800^1^ with 1800 cm^3^ of active volume. The response of this detector was calibrated using NIST standard reference x-ray beam qualities appropriate for the particular commercial scanners under study. During calibration, the IC was continuously and fully illuminated for a fixed time interval by the standard x-ray source. To contrast this with the different spatial and temporal illumination from an x-ray backscatter system, consider the x ray images shown in Fig. 3; these were separately acquired with image plates and subsequently superimposed. On the left is the Radcal IC used to measure incident air kerma from x-ray backscatter systems that were installed at NIST for extended study. The external (clamped) scales establish the distance between the concentric cylindrical electrodes; this 4.5 cm distance will be used later to estimate ion transit times. The x-ray image on the right is proportionally scaled to the IC and shows the results of exposing an image plate 30 cm from the beam-emitting surface (not the distance from the x-ray tube) of an AS&E SmartCheck X-ray Inspection System. This scanner employed an x-ray tube potential of 100 kV and one can see registration of individual tracks from the rastered pencil beam of x rays from a single-sided scan. The average of the pixel values for each row are plotted against pixel row number, for the indicated rectangular region of interest. The image plate was read off using the 300 dpi (dots per inch) setting; the area shown is therefore a relatively small fraction of the full scan area. As discussed below, these observations inform both how to perform the measurements of interest as well as how to interpret the results.

**Fig. 4:**
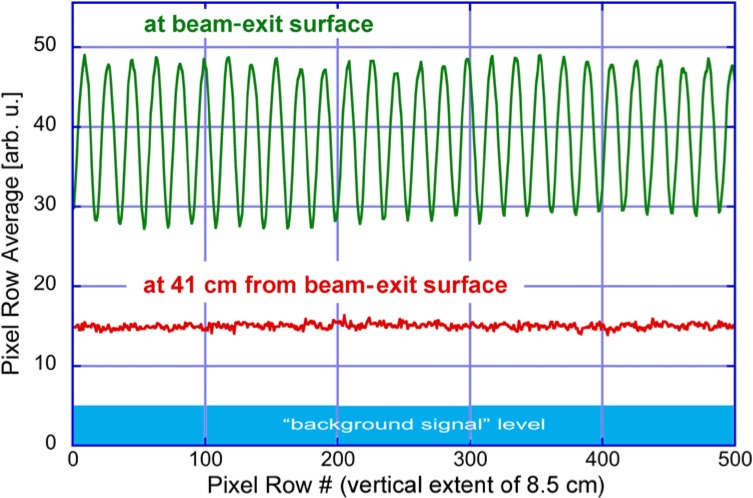
Analysis of image-plate data (similar to Fig. 2, right) due to single-scan exposures at 0 cm and 41 cm beyond the beam-exiting surface of a Rapiscan Secure 1000 Single Pose scanner that employed an x-ray tube potential of 50 kV.

To measure air kerma in a small, swept beam of x rays using a large-volume IC calibrated in a continuously- and fully-illuminated reference beam, one must be able to (1) operate the IC system in an integrating mode, (2) ensure that leakage current is negligible or accounted for over the integration time, (3) fully raster (“paint”) the IC as it would be during an operational scan, and (4) account for any incomplete charge collection due to incident fluence rates. The third point assumes that the pencil beam is smaller than the dimensions of the IC being used to measure air kerma (in our work, the pencil beam subtends less than a percent of the cross section of the IC). Partial volume corrections pertain only if part of the IC were blocked from the beam. If point (2) above is not satisfied, one must take additional care. One option is to employ specialized time-resolving electronics of the charge collection [11]. We did not find that such was needed for the commercial IC we used. A single-sided scan of the IC by a backscatter system produced integrated charge corresponding to tens of nGy while the background signal drift was limited to 0.02 nGy/s. Under conditions (1) to (3), the result of an IC air kerma measurement in a rastered x-ray beam is then correctly understood to be the *time integrated and spatially averaged* value over the area of the IC. The small-scale, spatial structure of the relative dose (as seen, *e.g.*, in the image plate data of Fig. 3) is a function of the distance from the source of the diverging pencil beam and the amount that the tails of each beam overlap with adjacent rasters as the source advances vertically. This variation with distance is clearly demonstrated in the plots of pixel-row averages in Fig. 4. Here image-plates were used to acquire single-scan exposures at 0 cm and 41 cm beyond the beam-exit surface of a Rapiscan Secure 1000 Single Pose scanner that employed an x-ray tube potential of 50 kV. We note that both the lateral dimensions of the pencil beam and its tangential velocity scale with the distance from an idealized point source; it follows that the pass-by time of the spot is very nearly independent of distance.

## 4. Air Kerma Measurements

While detailed dose maps through the inspection volume and estimates of absorbed-organ and whole-body doses are not the subject of this metrology paper, we report a single measurement of incident air kerma, primarily for the purpose of identifying typical components and magnitudes of uncertainty for this application. By way of illustration, we report the incident air kerma for the Rapiscan Secure 1000 Single Pose scanner that employed an x-ray tube potential of 50 kV. Both the Radcal 10X5-1800 ionization chamber and an RTI R100B solid-state detector (sensitive area 1 cm^2^) were calibrated using the NIST M50 standard beam quality [12]. The IC readings were also corrected for ambient temperature and pressure relative to standard environmental conditions. This “single pose” system uses two x-ray cabinets that face each other. The entire front and back sides of the body are rastered sequentially. For the measurement quoted here, the detectors were placed at a height of 1 m off the ground, and at 30 cm from the beam-emitting surface of the “front scanning” cabinet, and in the lateral center of the inspection volume. (The 30 cm distance is roughly the location of the front of an average-sized person, were they standing in the middle of the inspection volume.) The sum of the air kerma values delivered by the front and back scans was found to be 65.4 nGy ± 2.4 nGy [13] with about 70 % of this signal coming from the front scan since the IC was not in the center of the inspection volume. This reported value is an average of two measurements acquired after independent calibrations of the IC separated by four months and is assigned a relative expanded uncertainty of 3.6 % (coverage factor *k* = 2).

The foremost components of this uncertainty of the air kerma are given in Table 3 for Type A and Type B [14] uncertainties, and as discussed above, in this case an entry for leakage currents was not needed. In general an entry may also be required if the reference beam quality is sufficiently different (spectrally) from the source being measured, or needs to be extrapolated from two beam qualities, not the case here as discussed below. Because of its superior spatial sensitivity, the solid-state detector was primarily used to perform relative dose mapping measurements throughout the inspection volume. Nevertheless, it is interesting to note by comparison its post-calibration measurement result of the same exposure, 65.6 nGy ± 2 nGy, where this uncertainty simply reflects the statistical variation of repeated measurements at the same reference position. Such agreement from two such different detector systems helps give assurance that large systematics particular to a given technology are not being overlooked. Finally, it is noted that the response of this particular Radcal IC is factory-certified for a 150 kV x-ray spectrum with a half-value layer (HVL) of 10.2 mm of aluminum. When calibrated in the NIST M50 beam (50 kV; HVL = 1.04 mm Al), the calibration factor was determined to be 1.21. Hence, if calibration for this security application had been overlooked, the results would have been reported about 21 % too low.

Another critical measurement issue is the functional dependence of the incident air kerma with distance from the source. To compare the findings of different studies, or the exposures from different manufacturers, it is often desirable to extrapolate measurements of air kerma to a common position within the inspection volume. This obviously requires knowledge of the source position, and at least a relative mapping of air kerma in three dimensions. We have found that vendor-specific implementations of x-ray backscattering systems have resulted in dose distributions that differ from the inverse square law, as well as from each other. Such findings can be understood through fitting air kerma measurements as a function of source-to-detector distance and correlating the results to a particular type of source motion.

**Fig. 5:**
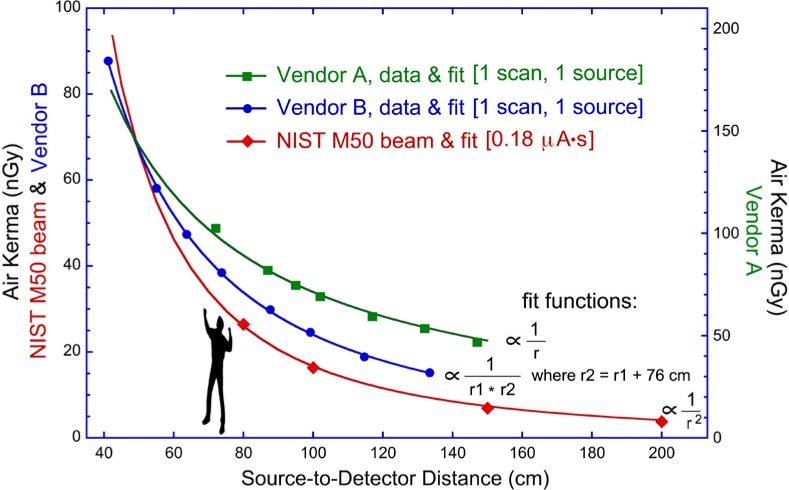
Functional dependence of absolute incident air kerma levels as a function of distance from the x-ray source for the NIST calibration source (M50 beam quality) and for two scanner implementations from different manufacturers of security x-ray backscatter systems. As discussed, the deviations of the scanner data from the inverse square law originate in the motion(s) of the sources. The scanner data were acquired by a Radcal 10X5-1800 IC with the appropriate calibration factors applied. The M50 beam (HVL = 1.04 mm Al) was measured by the Ritz national standard ionization chamber [12] during calibration of the Radcal IC.

Absolute air kerma measurements as a function of distance are shown in Fig. 5 for three different x-ray sources. The red data points were acquired using the same source (and therefore the same NIST M50 reference beam) that was used to calibrate the Radcal 10X5-1800 IC to the NIST M50 standard beam quality, a good proxy for the source represented with blue data points labeled Vendor B (Rapiscan Secure 1000 Single Pose scanner) [13]. The calibration data were recorded by the Ritz ionization chamber [12] positioned at the height of the fixed calibration source, as the stand off-distance is varied. The air kerma rate, and dose levels from the calibration source, exhibit the expected inverse square-law dependence with distance; these absolute rates have been scaled to the tube current (5 mA [6]) of Vendor B, and, in the absence of a direct measurement, the rates were multiplied by the pass-by time of the backscatter system of Table 2 (35 µs) to obtain the NIST air kerma values that are plotted. Distance-dependent air kerma is also plotted on the same (left) absolute vertical axis of Fig. 5 for the Vendor B source, as measured by the Radcal IC calibrated for this spectrum and at 1 m height from the floor, and in front of the laterally-centered x-ray tube. Finally, using the right-hand scale for the vertical axis, air kerma results are plotted for Vendor A (AS&E SmartCheck X-ray Inspection System) using the appropriate ionization chamber calibration factor for that system and similarly positioned in front of the source.

The two ordinate axes are employed to make apparent the different functional dependences of air kerma with distance. Manufacturers A and B have both implemented the lateral rastering scheme of Fig. 2. But each has implemented the vertical motion differently, and this accounts for the different functional fall off of signal with distance in the two cases as well as their difference from the inverse square law. In each case, the solid curves are fits to the data points shown. In the case of Vendor A, the x-ray tube is translated vertically during the course of a single scan. The cumulative effect is essentially that of a vertically translated fan beam, producing a radiation field that is found to fall off inversely with distance due to divergence primarily in one (the lateral) direction. The small vertical divergence within a single pencil beam is not the operative net effect within the inspection volume. While the physics is entirely different, perhaps it is helpful to note that the accumulated x-ray spatial pattern in the inspection volume, upon scanning a laterally-swept beam vertically without tilt, is analogous to the electric field-line pattern from an infinite vertical line charge, both fields varying inversely with distance (of course a similar analogy pertains for the inverse square nature of radiation/electric fields from point sources/charges). In recent models from Vendor A, there is a tilting motion at the bottom of a scan, to image the feet. In the case of Vendor B, however, the motion of the source is both vertically translated and tilted during the entire scan. The Vendor B data fit well to the inverse product of two different distances. One is the distance to the real source position due to the lateral motion as in the case of Vendor A; the second distance originates at the rotation axis of the vertical tilting motion, in this case about 76 cm *behind* the real source. To state the obvious, it is important to understand the system under study before making spatial extrapolations of air kerma values from commercial x-ray backscatter systems.

## 5. Estimates of Air Kerma Rate

Dose rates matter to radiobiology (not dealt with here), and air kerma rates matter to ionization chambers. Concerns have been expressed that since the x-ray pencil beams deliver their dose in such a brief pass-by time, the dose rates might be very large, even exceeding those of medical CT scans (this is not the case). To our knowledge, attempts to estimate the dose rates from these personnel-screening systems have not yet appeared in the literature, so we do so here, primarily to determine if they exceed the IC’s nominal detection rating, or more to the point, the IC’s ability to collect the saturation charge due to the swept-beam sources used in security screening. But first we establish empirically that the incident air kerma rates of the systems under study do not pose a metrology challenge to large, free-air ICs operated in integrating mode.

When using a free-air ion chamber for radiation dosimetry, readings must be corrected for incomplete charge collection, which primarily occurs when opposite charges, usually from different ionizing-particle tracks (in the case of general recombination), collide and recombine within the IC sensitive volume. Because recombination is a function of so many variables, including dose rate, it is recommended that collection efficiency be measured *in situ* using standard two-voltage techniques. The voltage-charge relationships for ionization chambers are modeled differently depending upon whether the incident radiation is continuous, pulsed, or swept [15]. All these formulations, however, assign negligible recombination to the case where the same quantity of charge is collected at two widely different bias voltages and at constant dose rate. (In general, other sources of incomplete collection efficiency can exist that are not captured by the two-voltage technique; in view of subsequent discussion, they are not considered important for the present application.)

The two-voltage technique was applied with the Radcal 10X5-1800 ionization chamber positioned at the same reference position described in the previous section and in front of the same Rapiscan single-pose scanner. The IC integrated front and back sequential scans, as per normal-use screening; measurements were obtained for six trials and averaged. To within statistical uncertainties, the charge collected was 3.53 pC at both 300 V (routine IC operating potential) and 150 V. At 75 V it had dropped by 1 %, with further losses at lower potentials. Hence, normal operation of this IC is found to fall within the saturation region, and charge collection is near unit efficiency under irradiation by the rastered pencil beam of this screening system.

The most obvious method for estimating air kerma rate would be to divide a measured air kerma reading by the pass-by time of the pencil beam of x rays. This however requires both an accurate measurement or knowledge of the pass-by time (and in general the temporal profile), as well as an air kerma measurement of a single pass of a single pencil beam. All of our air kerma measurements are integrated over a scan and return a value that includes contributions from the spatially-overlapping tails of nearby rasters. While this returns the dose level of interest for estimating absorbed dose to persons, it is not identical to the value from a single pass of the pencil beam. Alternatively, we estimate the dose rate of the Rapiscan Secure 1000 SP system using two independent methods outlined below. But first we observe that while the incident air kerma vs. distance from the source differs among security screening systems (Fig. 5), the air kerma *rate* at a point in the inspection volume from a single pencil beam should only be a function of the source spectrum, tube current, and distance from the source to the point of measurement. The variety of distance dependences seen in Fig. 5 for the integrated air kerma levels originated in the motion(s) of the x-ray source during a scan. That air kerma rate is independent of source motion follows from the previous observation that the pencil beam is produced merely by vignetting a conventional x-ray tube, with no compression in time or focusing in space.

**Fig. 6:**
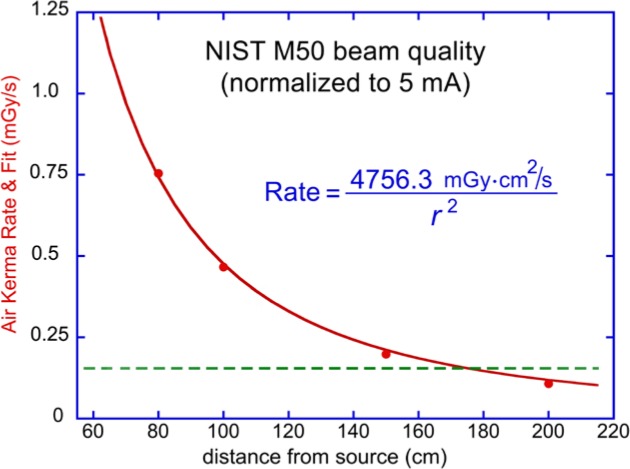
Absolute air kerma rate of the NIST M50 beam quality as a function of distance from the source. The curve is an inverse-square fit to measurements (circular plot symbols) acquired by the NIST Ritz free-air ionization chamber, a primary national standard for x-ray beams between 20 kV and 100 kV [12]. The data were acquired at 2 mA tube current, and normalized to 5 mA as a proxy for the Rapiscan Secure 1000 Single Pose scanner [6]. The dotted horizontal line indicates the nominal rate limit of the Radcal 10X5-1800 ionization chamber in a continuous beam and full spatial illumination, 0.16 mGy/s.

As aforementioned, the NIST M50 standard beam quality has a spectrum very similar to the Rapiscan system under discussion. The absolute air kerma rate of the former was measured during the calibration of the detectors used in our studies. Figure 6 shows determinations of air kerma rate at four distances, along with a fit to the inverse square law. The fit equation shown in Fig. 6 was used to estimate the air kerma rate produced by a flying spot in the Rapiscan system. At a distance of 75 cm the air kerma rate is estimated to be 0.85 mGy/s using this method. The data shown in Fig. 6 were acquired by the NIST Ritz free-air ionization chamber, a primary national standard instrument for x-ray beams between 20 kV and 100 kV [12]. The values shown were acquired with 2 mA tube current, and normalized to 5 mA as a proxy for the Rapiscan Secure 1000 Single Pose scanner [6]. These Ritz IC data are numerically equal to the *calibrated* Radcal IC data shown in Fig. 5 for the M50 beam (before time and current scaling). Alternatively, for a sufficiently characterized source, one can estimate air kerma rates from first principles. Using the IPEM Report 78 spectrum generator [16] and our measured HVL of the Rapiscan system of 1.085 mm of Al [13], a value of 0.85 mGy/s is obtained for the air kerma rate at a distance of 75 cm from the source (obtained by multiplying the value of 169.3 µGy/mA·s from the IPEM generator by the Rapiscan tube current of 5 mA). The agreement obtained between the two methods described above for estimating the air kerma rate value of 0.85 mGy/s is surely fortuitous due to various idealizations in these two independent methods of estimation; nevertheless, the air kerma rate plot in Fig. 6 is estimated to be a good representation for the Rapiscan output ± 5 % (1σ). For comparison with medical dose rates, if one applies the IPEM Report 78 model to the case of medical CT scans (with these typical parameters: tungsten anode, HVL = 7.5 mm of Al, 200 mA, tube voltage = 120 kV), then at the same 75 cm stand off, 25.8 mGy/s is obtained, a factor of about 30 more than the air kerma rates from backscatter systems. (This should not be confused with the absolute dose to persons by security backscatter systems [8,13] that has been measured to be many orders of magnitude less than medical procedures.)

The dotted line in Fig. 6 indicates the vendor’s stated nominal air kerma rate limit of the Radcal 10X5-1800 ionization chamber, 0.16 mGy/s. This is lower than the values on the fit curve for most measurement locations in the inspection volume of x-ray backscatter systems. The use of large-volume air ion chambers has been questioned due to this apparent violation of the vendor’s stated dose-rate limit [17]. This limit applies, however, to a temporally continuous beam with illumination that spatially overfills the IC, which is obviously not the case in the application dealt with here. Incident air-kerma rate per se is not really the issue, but whether ion recombination is occurring and accounted for. To help explain the lack of measureable recombination during backscatter metrology, the underlying physics is discussed below.

The adequacy of any dosimetric method requires consideration of both the properties of the source and the dosimeter. The case of a rastered spot of x rays presents a source that is unusual in both time and space compared to most applications of radiation dosimetry. An ionization chamber’s response depends upon the rate at which charge is produced and the ability to collect such free charge before it can be lost, mainly through recombination with overlapping clouds of the opposite charge. In our system, positive ions are collected after transiting through free air. The transit time in turn is a function of chamber geometry, electrode gap distance, and applied voltage. Some simple measurements and calculations characterize the regime into which the present application typically falls.

**Fig. 7:**
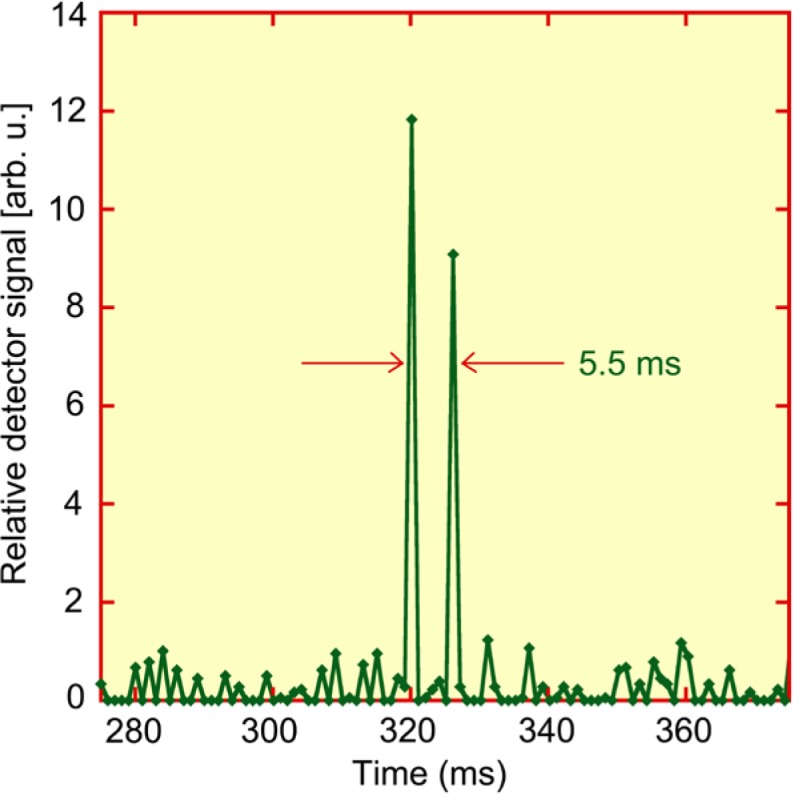
Sweep time measured at 1 m height and 30 cm from the beam-exiting surface of a Rapiscan Secure 1000 Single Pose scanner. The pencil beam of x rays from consecutive horizontal sweeps was registered with an RTI CT Dose Profiler (solid state) with 1 ms resolution. The average of 21 measurements gave 5.5 ms ± 0.1 ms (*k* = 1).

For illustration, consider the arrangement discussed above, *viz.*, the Radcal IC placed 30 cm in front of the beam-exiting surface of one of the two Rapiscan cabinets. The IC intercepts about one seventh of the lateral extent of the horizontal motion of the pencil beam. So during a normal scan, in the time domain at least, the radiation is pulsed and “on” about 14 % of the time. If fully illuminated (spatially), one might expect recombination equivalent to that of a continuous beam with same mean dose rate, *i.e.*, 14 % of the values along the curve in Fig. 6. To measure the pulse spacing, we replaced the IC with a solid-state RTI CT Dose Profiler. Figure 7 shows the detection in time of two consecutive transits of the flying spot, equivalent to the time of a single horizontal sweep of the pencil beam. The detector’s sensitive volume is about 3 mm in the vertical direction of the source motion, so depending upon registration, some scans exhibited three or four transits, albeit with signals of varying intensity, and all separated by about the same time interval. The average interpulse spacing was found to be 5.5 ms ± 0.1 ms, where the uncertainty quoted is the sample standard deviation of 21 repeated trials. (The 1 ms instrumental resolution did not permit determination of the width or time structure of an individual pulse.) It is also straightforward to estimate the transit time, *T*, for the slowest ions produced by each pulse to be collected [18] within the Radcal IC.

$$T={\left\{(a-b)K_{\mathrm{cyl}}\right\}}^{2}/Vk={\left\{(4.5\;\mathrm{cm})(1.06)\right\}}^{2}/(300V)(1.3\;{\mathrm{cm}}^{2}s^{-1}V^{-1})\approx 58\;\mathrm{ms}$$

where a − b is the difference of inner and outer concentric cylindrical electrode radii, taken from Fig. 3, *K*_cyl_ = 1.06 is a field distortion factor for a cylindrical ion chamber, itself a function of *a* and *b*, *V* = 300 V is the applied voltage during charge collection, and *k* is the mean (positive) ion mobility for free air, taken to be 1.3 cm^2^s^−1^V^−1^ [18]. The ratio of the calculated transit time and measured pulse spacing is 58 ms/5.5 ms ≈ 10. Hence for the conditions modeled here, there will be about ten temporally-overlapping pulses of ionization in transit between the nested cylindrical electrodes within the IC during most of the exposure time. And since during a typical scan a large IC will be vertically “painted” with dozens of horizontal sweeps of the pencil beam (see Figs. 3 and 4), most of the volume of the ion chamber will not be actively producing charge during the scan. These temporal and spatial differences which are far from continuous and full illumination both tend to greatly reduce the likelihood of ion recombination, despite an in-beam dose rate that is larger than the nominal IC limit. Such analysis offers some physical understanding underlying the measurement result of negligible recombination from the *in situ* two-voltage technique applied to this screening system and ionization chamber combination.

## 6. Conclusions

In this paper we have assessed the tools and methods recently recommended (Table 1, columns 2 and 3) for the metrology of a rastered spot of x rays, motivated by their recent use in the screening of persons for non-medical purposes. While the x-ray sources employed are somewhat uncommon, it is found that the common tools already in use in radiation dosimetry, properly applied and interpreted, are entirely adequate to the task of accurately determining incident air kerma levels and estimating air kerma rates for these security-screening systems (AIT). Knowledge of spectra and incident air kerma values from x-ray backscatter systems permits the estimation of organ and whole-body doses as would then be undertaken for any other source of ionizing radiation. Finally, accounting for the various angular illumination patterns that result from these moving and rastering sources (Fig. 5) can enhance the accuracy of models that are used to estimate doses to persons from these security-screening systems.

## Figures and Tables

**Table 1:** Standards and Guides for X-Ray Advanced Imaging Technology (AIT)

Imaging Performance Standards	Radiation Safety Standards	Guides
		
ANSI N42.47 – 2010 [1]	ANSI/HPS N43.17 – 2009[3]	NCRP Commentary 16 [5] AAPM Report 217 [6]
IEC 62709 – 2014 [2]	IEC 62463 – 2010 [4]	SCENIHR – April 2012 [7]

**Table 2:** Example specifications for a rastered x-ray backscatter system for personnel screening

X-ray spectrum:	Tungsten target, 20° anode angle, filtration 1 mm Al equivalent, 50 kV potential
Focal spot size:	1 mm
Tube current:	5 mA
Geometry:	- center of inspection area: 877 mm from focal spot - beam size at 877 mm: 5.5 mm × 5.5 mm - width of horizontal sweep: 1000 mm - x-ray beam horizontal sweep time: 5.45 ms - field moving up 4.82 mm during each horizontal sweep - each location (at one sweep) exposed approximately 35 μs - total scan height: 2.3 m
Duration of each scan:	2.6 s
Tilt at top/bottom:	± 45° (not exactly specified)

Front scan followed by back scan at same conditions

**Table 3:** Air kerma uncertainty budget for calibration of Radcal 10X5-1800 ionization chamber and its use with an x-ray backscatter security scanner (relative uncertainties in %)

Uncertainty Components	Type A	Type B
***Ionization Chamber Calibration @ 2 m from source***		

reference air kerma rate	0.19	0.37

air kerma measurement in reference beam	0.10	0.90

air density correction	0.01	0.08

effective measurement point		0.10

positioning		0.10

recombination	0.10	

quadratic sum	0.24	0.99
combined uncertainty chamber calibration	1.01	

***Air Kerma Measurement of a Rapiscan Secure 1000 SP***		

chamber calibration (repeated from above)	0.24	0.99

air kerma measurement in scanner beam	0.25	

reproducibility of air kerma measurement (includes positioning)		1.00

ambient pressure		1.00

ambient temperature		0.20

IC illumination different from calibration: (a) finite size of the IC and use at diff distances (b) scanner illumination diff from inverse-square law		0.3

		0.3

quadratic sum	0.34	1.79
total combined uncertainty (*k* = 1)	1.8	
total expanded uncertainty (*k* = 2)	3.6	

## Footnotes

1 Certain commercial equipment, instruments, or materials are identified in this paper in order to specify the experimental procedure adequately. Such identification is not intended to imply recommendation or endorsement by the U.S. government, nor is it intended to imply that the materials or equipment identified are necessarily the best available for the purpose.
